# Sulfur-SAD phasing from microcrystals utilizing low-energy X-rays

**DOI:** 10.1107/S2052252519008698

**Published:** 2019-06-28

**Authors:** Zbigniew Dauter

**Affiliations:** a National Cancer Institute, Argonne National Laboratory, Argonne, IL 60439, USA

**Keywords:** S-SAD, native SAD, microcrystals, microdiffraction, radiation damage, multiple crystals, anomalous diffraction, low-energy X-rays

## Abstract

A practical approach for obtaining S-SAD data from native protein microcrystals with low-wavelength synchrotron radiation [Guo *et al.* (2019), *IUCrJ*, **6**, 532–542] is presented in this issue of **IUCrJ**.

The utility of the weak anomalous signal provided by the low-*Z* atoms of sulfur (in proteins) or phospho­rus (in nucleic acids) was recognized already in the early days of macromolecular crystallography. The anomalous difference peaks served as anchors, helping to locate cysteine and me­thio­nine residues (or phosphates) during the building of initial models for proteins or nucleic acids within electron density maps. However, the further possibility of actively using such signals for the phasing of macromolecular structures was documented in the 1980s by Hendrickson & Teeter (1981 ▸) and Wang (1985 ▸).

Nevertheless, the practical use of the anomalous signals from low-*Z* atoms for phasing had to await the improvement of diffraction data accuracy resulting from the introduction of stable synchrotron beamlines, automatic 2D detectors and powerful phasing algorithms. These developments led to the popularization of the single-wavelength anomalous diffraction (SAD) phasing technique. SAD phasing is the only method applicable in these cases, since the S and P atoms have their X-ray absorption edges at 5.02 and 5.78 Å, thus are not practically amenable for MAD. On the other hand, the anomalous signals of these atoms increase with X-ray wavelength and their *f*′′ values approach 1.0e for λ close to 2.5 Å. Beyond that point, however, absorption effects preclude the practical use of such long wavelengths.

The serious challenge to utilization of the very weak anomalous signal is the inevitable radiation damage of macromolecular crystals by the absorbed X-ray quanta. To circumvent this problem, Hendrickson and colleagues (Liu *et al.*, 2011 ▸) proposed to merge data from multiple crystals, and this serial approach prompted creation of dedicated algorithms and programs for optimal selection and merging of data from many diffraction images (*e.g.* Foadi *et al.*, 2013 ▸; Guo *et al.*, 2018 ▸).

Another tendency in macromolecular crystallography common in recent years is the development of techniques for collecting diffraction data from microcrystals with dimensions of 10 µm or even smaller. This was made possible by serial femtosecond crystallography (SFX; Chapman *et al.*, 2011 ▸) with the use of the X-ray free electron laser sources (XFELs), where a slurry containing the microcrystals is injected into the super-brilliant X-ray beam. Each microcrystal targetted provides only one diffraction image before the crystal is completely destroyed and therefore this approach requires recording and processing of hundreds of thousands of images, with the majority of them being ‘empty’. A sulfur-SAD (S-SAD) experiment using SFX is very complicated, time consuming, and not easily accessible for non-specialized users.

This issue of **IUCrJ** contains an article (Guo *et al.*, 2019 ▸) published by a team of crystallographers from Brookhaven National Laboratory, NY, and Columbia University, NY (including the guru of anomalous diffraction, Wayne Hendrickson), where the more practical approach to collect data from microcrystals is proposed. Their paper describes in considerable detail a successful S-SAD phasing experiment with data obtained at a standard contemporary synchrotron beamline using microcrystals of thaumatin at a wavelength of 2.48 Å, using only about 1200 crystals.

The microcrystal slurries were loaded on low-absorption polyimide mesh well mounts that were then dipped in liquid nitro­gen. The FMX beamline at NSLS-II was equipped with an Eiger 16M detector and the beam was collimated to 5 × 9 µm. The helium chamber was not used and the X-ray beam was not excessively intense, but the sample absorption was estimated to be only about 2%. The best spatial resolution was 2.6 Å and the meaningful anomalous signal was observed to a spatial resolution of between 3.5 and 4.2 Å (Fig. 1 ▸).

The experiment resulted in 1381 partial data sets obtained from 18 well mounts. The final data set was assembled by merging the data from carefully selected individual crystals and images, with the rejection of more problematic frames. After a careful post-mortem examination of various frame rejection schemes, it transpired that the most beneficial rejection criteria for S-SAD phasing should not be very stringent, as a compromise between data multiplicity, radiation damage and other effects.

The approach described by Guo *et al.* opens the way for practical and effective solution of crystal structures for macromolecules that do not form large crystals, do not diffract to high resolution, and are not amenable for derivatization, such as many membrane proteins or multi-molecular complexes. In addition, as stated in the paper, inclusion of the helium environment would significantly facilitate such S-SAD experiments.

## Figures and Tables

**Figure 1:**
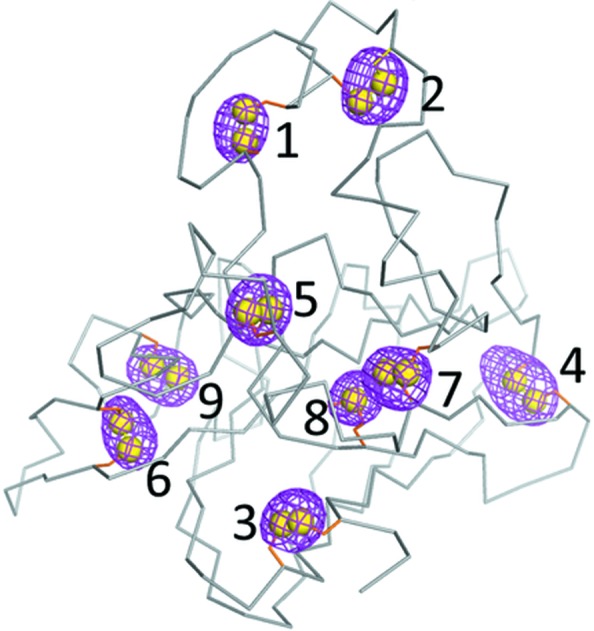
Anomalous difference peaks identified in thaumatin from the S-SAD data.. Reproduced from Guo *et al.* (2019 ▸).
